# Thalidomide plus prednisone with or without danazol therapy in myelofibrosis: a retrospective analysis of incidence and durability of anemia response

**DOI:** 10.1038/s41408-017-0029-4

**Published:** 2018-01-15

**Authors:** Xueping Luo, Zefeng Xu, Bing Li, Tiejun Qin, Peihong Zhang, Hongli Zhang, Liwei Fang, Lijuan Pan, Naibo Hu, Shiqiang Qu, Yue Zhang, Gang Huang, Robert Peter Gale, Zhijian Xiao

**Affiliations:** 1 MDS and MPN Centre, Institute of Hematology and Blood Diseases Hospital, Chinese Academy of Medical Sciences and Peking Union Medical College, Tianjin, China; 2 State Key Laboratory of Experimental Hematology, Institute of Hematology and Blood Diseases Hospital, Chinese Academy of Medical Sciences and Peking Union Medical College, Tianjin, China; 3 Department of Pathology, Institute of Hematology and Blood Diseases Hospital, Chinese Academy of Medical Sciences and Peking Union Medical College, Tianjin, China; 4 Divisions of Experimental Hematology and Cancer Biology, Cincinnati Children’s Hospital Medical Center, Cincinnati, OH USA; 5 Haematology Section, Division of Experimental Medicine, Department of Medicine, Imperial College London, London, UK

## Abstract

Low-dose thalidomide and prednisone alone or combined are effective therapies in some persons with primary myelofibrosis (PMF) and anemia with or with RBC transfusion dependence. Danazol is also effective in some persons with PMF and anemia. Responses to these drugs are typically incomplete and not sustained. It is unclear whether adding danazol to thalidomide and prednisone would improve efficacy. We retrospectively compared the outcomes of 88 subjects with PMF and anemia receiving thalidomide and prednisone without (*n* = 46) or with danazol (*n* = 42). The primary end point was anemia response, which was 71% (95% confidence interval (CI), 57, 85%) in subjects receiving thalidomide/prednisone/danazol compared with 46% (32, 60%; *P* = 0.014) in those receiving thalidomide/prednisone. Response rates in subjects who were RBC transfusion dependent was also higher in the danazol cohort (61% (38, 84%)) vs. 25% (6, 44%); *P* = 0.024). Time to response was rapid (median, 2 months (range, 1–11 months)) and similar between the cohorts. Response duration was longer in the thalidomide/prednisone/danazol cohort (HR 2.18 (1.18–5.42); *P* = 0.019). Adverse effects were mild and similar between the cohorts. In conclusion, thalidomide/prednisone/danazol seems superior to thalidomide/prednisone in persons with PMF and anemia. Our conclusion requires confirmation in a randomized trial.

## Introduction

About one-third of persons with primary myelofibrosis (PMF) have anemia at diagnosis and it develops in most others as the disease evolves^1,2^. Anemia and red blood cell (RBC) transfusion dependence are independent adverse prognostic variables for survival^1–3^. Erythropoiesis-stimulating agents (ESAs), androgenic steroids, thalidomide, lenalidomide, splenectomy, and prednisone are only modest activity in reversing anemia and ruxolitinib, pacritinib and fedratinib typically worsen anemia^4–7^. New effective therapies are needed.

Thalidomide is active in PMF because of its anti-angiogenic, cytokine regulatory, and immune-modulating properties^8,9^. Thalidomide, 100–400 mg/day, is reported to improve anemia in 20–60% of subjects^10–12^. However, these doses are associated with substantial toxicity and are poorly tolerated^10,11^. The combination of low-dose thalidomide, 50 mg/day, and prednisone is better tolerated and results in slightly higher responses than thalidomide alone^13^.

Androgenic steroids reverse anemia by stimulating erythropoietin, increasing iron use and reversing telomere loss^14,15^. Danazol, a synthetic androgen with reduced masculinizing activity, is reported to reverse anemia and thrombocytopenia in persons with PMF^16,17^. Combining therapies with different mechanisms of action might be even more effective in reversing anemia. Herein, we report efficacy, safety, and long-time outcome of therapy with low-dose thalidomide and prednisone with or without danazol in subjects with PMF and anemia with or without RBC transfusion dependence.

## Subjects and methods

### Subjects

This study was approved by the Ethical Committee of Institute of Hematology, CAMS and PUMC according to guidelines of the Declaration of Helsinki. From March 2006 to September 2016, 88 consecutive subjects and anemia with or without RBC transfusion dependence were enrolled. Eligibility criteria were: (1) PMF according to the WHO 2016 criteria^18^; (2) age ≥ 18 years; (3) hemoglobin concentration <100 g/L or RBC transfusion dependence^19^; (4) no exposure to ESAs, androgens, thalidomide, lenalidomide, or corticosteroids <12 weeks pre-enrollment; (5) creatinine ≤2 mg/dL, direct bilirubin <2 times upper limit of normal (ULN) and alanine aminotransferase (ALT)/aspartate aminotransferase (AST) ≤3 ULN. Subjects receiving ruxolitinib during the past several years were excluded. Subjects had a pre-therapy physical examination, baseline laboratory assessment of serum chemistries and blood hematologic parameters, bone marrow aspirate and biopsy, and cytogenetic analyses. Prognostic cohort was assigned using the Dynamic International Prognostic Scoring System (DIPSS)^2^ for all subjects and DIPSS-plus^3^ for those with cytogenetics data. Bone marrow fibrosis was graded using European consensus guidelines^20^.

### Therapy

Subjects received thalidomide, 50 mg p.o. at bed time continuously. Prednisone, 0.5 mg/kg/day, was given for 1 month, 0.25 mg/kg/day, for the next month, 0.125 mg/kg/day for the third month and tapered thereafter. Danazol, 600 mg/day p.o. was given continuously. Patients with neutrophil count <1.0 × 10E + 9/L and/or platelet count <80 × 10E + 9/L were assigned to thalidomide/prednisone/danazol therapy, the others were assigned to thalidomide/prednisone therapy. Laboratory studies were performed weekly for 12 weeks. Responders continued on their assigned therapy, whereas others stopped. Packed RBCs were transfused for a hemoglobin concentration <60 g/L or symptoms of anemia.

### Evaluation of response and adverse events (AEs)

The primary study outcome was anemia response. In subjects with splenomegaly or thrombocytopenia, spleen and platelet responses were also analyzed. Anemia and spleen responses were assessed according to the revised International Working Group for Myelofibrosis Research and Treatment (IWG-MRT) consensus criteria^21^. Thrombocytopenia response was defined as a platelet increase >50 × 10E + 9/L in subjects with baseline platelets <100 × 10E + 9/L. Toxicity was assessed by the National Cancer Institute Common Toxicity Criteria for Adverse Events, Version 4^22^.

### Statistical analyses

Follow-up was to death or 10 June 2017. Quantitative data are expressed as median and range and qualitative data as percent. Baseline variables were compared between cohorts using χ^2^ test for categorical variables and Mann–Whitney *U*-test for continuous variables. Clinical responses were compared between cohorts with χ^2^ test. Logistic regression was used to assess relationships between baseline variables and outcomes. Variables significant in univariate analyses were included in the multivariate analysis. Response duration was defined as the interval from anemia response to loss of response, change of therapy, or death. Response duration was calculated using the Kaplan–Meier method and compared by the log-rank test. *P*-values are two-sided and statistical significance defined as *P* < 0.05. Statistical analyses were performed using the IBM SPSS 22.0 package (SPSS, Chicago, IL, USA).

## Results

### Subject- and disease-related variables

Eighty-eight subjects with PMF and anemia were evaluated. Forty-six received thalidomide and prednisone and 42 received thalidomide, prednisone, and danazol. Baseline variables were similar (Table 1). Median age was 53 years (range, 21–77 years). Forty-six (52%) were male. Thirty-eight subjects (43%) were RBC transfusion dependent^19^ and 49 (56%) had platelets <100 × 10E + 9/L. Median hemoglobin concentration and median white blood cell (WBC) and platelet levels were 71 g/L, 3.49 × 10E + 9/L and 84 × 10E + 9/L, respectively. Spleens were palpable a median of 4 cm (range, 0–24 cm) below the left costal margin (LCM). Fourteen of 54 subjects (26%) with evaluable cytogenetics had unfavorable karyotypes according to DIPSS-plus^3^. Thirty-one of 70 subjects tested had *JAK2*^V617F^. Forty-five subjects (51%) were intermediate-1, 38 (43%) were intermediate-2 and 5 (6%), high-risk according to the DIPSS^2^.

**Table 1:** Baseline characteristics of patients

Variable	Thalidomide/prednisone	Thalidomide/prednisone/danazol	*P*-value
No.	46	42	
Age (years)	53 (21, 77)	53 (26, 72)	0.776
Male *n* (%)	24 (52.2%)	22 (52.3%)	0.985
Previous therapy	13 (28.3%)	18 (42.9%)	0.152
Spleen size below left costal margin (cm)	3 (0,18)	5 (0, 24)	0.606
Constitutional symptoms	18 (39.1%)	11 (26.2%)	0.197
Hemoglobin (g/L)	71 (31, 99)	71 (38, 99)	0.854
WBC counts (×10^9^/L)	4.07 (0.75, 22.36)	3.22 (1.51, 25.63)	0.335
Platelets counts(×10^9^/L)	100 (3, 1064)	80 (14, 540)	0.631
Serum EPO (mU/mL)	504 (21, 774)	652 (76, 774)	0.845
RBC transfusion dependency	20 (43.5%)	18 (42.9%)	0.953
Blood blasts%	0 (0,17)	0 (0, 2)	0.100
Marrow blasts%	0 (0,7)	0 (0,5)	0.202
Bone marrow fibrosis			0.328
MF-2	35 (76.1%)	28 (66.7%)	
MF-3	11 (23.9%)	14 (33.3%)	
Cytogenetics^a^			0.777
Unfavorable	9 (27.3%)	5 (23.8%)	
Favorable	24 (72.7%)	16 (76.2%)	
*JAK2*^V6A7F^ mutation^b^			0.810
Positive	16 (46%)	15 (43%)	
Negative	19 (54%)	20 (57%)	
DIPSS risk group			0.217
Intermediate-1	20 (43.5%)	25 (59.5%)	
Intermediate-2	22 (47.8%)	16 (38.1%)	
High	4 (8.7%)	1 (2.4%)	

*WBC* white blood cell, *EPO* erythropoietin, *DIPSS* Dynamic International Prognostic Scoring System

^a^Cytogenetic information was available in 54 patients

^b^*JAK2*^V6A7F^ mutation status was available in 70 patients

Thirty-one subjects received prior therapy(ies) including 9, thalidomide; 20, androgenic steroids; 5, recombinant human erythropoietin; 3, corticosteroids; 4, hydroxyurea; 3, interferon; and 1, melphalan. Median interval from diagnosis to study entry was 0 month (range, 0–62 months). Althought on-study, nine subjects received hydroxycarbamide (hydroxyurea) alone (*N* = 7) or with interferon (*N* = 2) and two received melphalan.

### Responses and outcome

The anemia response rate for all subjects was 58% (95% confidence interval (CI) 48, 68%). Subjects receiving thalidomide/prednisone/danazol had a significantly higher response rate compared with those receiving thalidomide/prednisone (71% (57, 85%) vs. 46% (32, 60%); *P* = 0.014). Response rates in subjects who were RBC transfusion dependent were also higher in the danazol cohort (61% (38, 84%) vs. 25% (6, 44%); *P* = 0.024).

There is no significant correlations between anemia response rate and *JAK2*^V17F^ mutation state (*P* = 0.238). Sixty-eight percent (52, 84%) of *JAK2*^V617F^ subjects responded compared with 54% (38, 70%) JAK2 wild-type subjects (*P* = 0.238). In subgroup analysis, 63% (39, 87%) of *JAK2*^V617F^ subjects responded compared with 42% (20, 64%) JAK2 wild-type subjects among patients receiving thalidomide/prednisone (*P* = 0.229), 73% (51, 95%) of *JAK2*^V617F^ subjects responded compared with 65% (44, 86%) JAK2 wild-type subjects among patients receiving thalidomide/prednisone/danazol (*P* = 0.875).

In multivariate analyses, only thalidomide/prednisone/danazol therapy (odds ratio (OR) = 3.39 (1.29, 8.89); *P* = 0.013) and not being RBC transfusion dependent (OR = 2.90 (1.11, 7.61); *P* = 0.03) were significantly associated with response (Table 2). Responses occurred rapidly: median time to response was 2 months (range, 1–11 months) and did not differ between the cohorts. Interval to response varied: 61% of responders did so by 3 months, 94% by 6 months and only 6% after 6 months.

**Table 2:** Multivariate analysis of anemia response

Variable	OR (95% CI)	Multivariate analysis, *P*
Treatment group		0.013
Thalidomide/prednisone	1	
Thalidomide/prednisone/danazol	3.39 (1.29–8.89)	
Gender		0.161
Male	1	
Female	2.01 (0.76–5.31)	
RBC transfusion dependent		0.03
≥6U/12W	1	
<6U/12W	2.90 (1.11–7.61)	
Palpable spleen length		0.225
LCM <5 cm	1	
LCM ≥5 cm	1.82 (0.69–4.79)	

*OR* odds ratio, *CI* confidence interval,* LCM* left costal margin

**Fig. 1:**
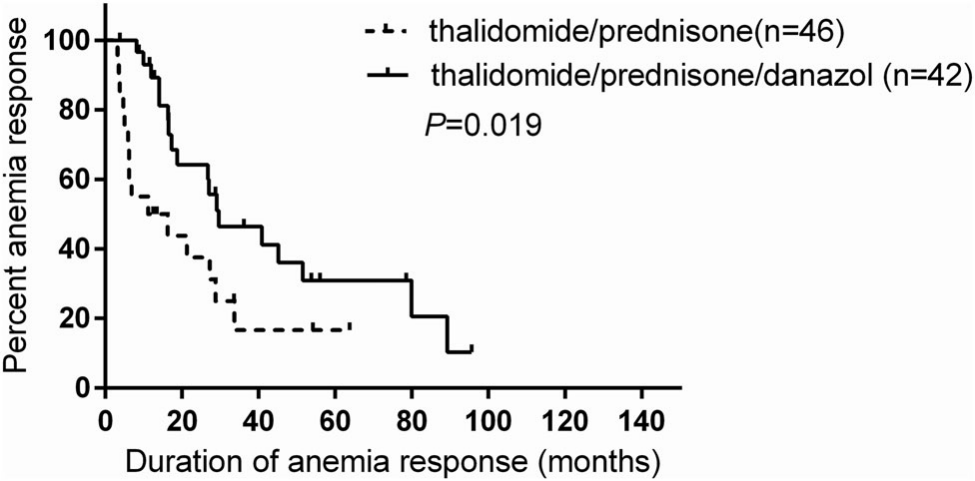
Response duration according to the treatment groups

The minimum duration of treatment was 3 months, with a median of 25 months (range 3–117+ months). Subjects receiving thalidomide/prednisone/danazol had significantly longer response durations than those receiving thalidomide/prednisone (hazard ratio (HR) 2.18, 95%CI (1.18–5.42), *P* = 0.019; Fig. 1). Median response duration was 27 months (95% CI, 15–39 months) overall, 30 months (10–49 months) in the thalidomide/prednisone/danazol cohort compared with 11 months (0–30 months) in the thalidomide/prednisone cohort.

Twenty-two of 49 subjects (45% (31, 59%)) with baseline platelets <100 × 10E + 9/L had an increase of >50 × 10E + 9 L including 58% (39, 77%) of subjects receiving thalidomide/prednisone/danazol vs. 30% (11, 49%; *P* = 0.06) of subject in the thalidomide/prednisone cohort. Median time to platelet response was 3 months (range, <1–9 months) and was similar between the cohorts as was response duration (21 months (95% CI, 7–35 months)). There was a spleen response in 16 of 41 evaluable subjects (39% (24, 54%)) with similar response rates between the cohorts.

### Adverse events

AEs were dose dependent and reversible with similar incidences (save ALT/AST increases) and severities in the cohorts. No subject discontinued therapy because of drug-related AEs. Leukocytosis and thrombocytosis occurred in 19% (11, 27%) and 24% (15, 33%) of subjects. There was no thrombo-embolic event. The most frequent non-hematologic AE was increased ALT/AST in 19% (8, 31%) of subjects receiving thalidomide/prednisone/danazol compared with 4% (3, 22%; *P* = 0.07) of subjects receiving thalidomide/prednisone. Other non-hematologic AEs were less frequent and did not differ significantly between the cohorts (Table 3) including increased bilirubin in four, constipation in six, hyperglycemia in six; rash in five; edema in six, neurological symptoms in seven; abdominal distention in four; hypertension in three, somnolence in two, and creatinine elevation in two. There was no case of prostate cancer or hepatic adenoma.

**Table 3:** Toxicity of the therapeutic regimen

Adverse events	Thalidomide/prednisone (*n*=46)	Thalidomide/prednisone/danazol (*n*=42)
Hyper-bilirubinemia	1	3
Constipation	4	2
Edema	3	3
Hyperglycemia	3	3
Rash	3	2
Neurological symptoms	5	2
Abdominal distention	2	2
Hypertension	1	2
Somnolence	2	0
Creatinine elevation	0	2

## Discussion

Efficacy of low-dose thalidomide/prednisone in persons with PMF and anemia was studied in relatively small series of subjects with variable response criteria and response rates^13,23,24^. We confirmed the efficacy of thalidomide/prednisone using the current IWG-MRT criteria^21^. Importantly, adding danazol significantly increased response. In another recent study, danazol alone was reported to have a response rate of 30% (17, 43%)^17^^.^ These data suggest therapy with thalidomide/prednisone/danazol is better than any component therapy.

Previous studies reported lower serum EPO levels, smaller spleen size, and lower RBC transfusion frequency were associated with higher anemia response rates^17,25,26^. We found such an association only for RBC transfusion dependence, and independence. We also found an advantage for combined therapy in subjects who were RBC transfusion dependent. Anemia responses occurred quickly and similarly in the two groups. Median time to response was shorter in our study than a previous study of danazol^17^ suggesting thalidomide/prednisone may have accelerated danazol responses.

Our data suggest adding danazol to thalidomide/prednisone improves response rates, prolongs response duration in persons with PMF and anemia with and without RBC transfusion dependence. The major limitation of our study is that it was a retrospective analysis and not randomized, and that although the cohorts were similar for known predictive variables we cannot be certain they were comparable for unknown predictive variables. As such, our conclusions require confirmation in a randomized trial.
